# Granulocyte-Colony Stimulating Factor Attenuates Oligomeric Amyloid β Neurotoxicity by Activation of Neprilysin

**DOI:** 10.1371/journal.pone.0103458

**Published:** 2014-07-25

**Authors:** Yukiko Doi, Hideyuki Takeuchi, Hiroyuki Mizoguchi, Kazuya Fukumoto, Hiroshi Horiuchi, Shijie Jin, Jun Kawanokuchi, Bijay Parajuli, Yoshifumi Sonobe, Tetsuya Mizuno, Akio Suzumura

**Affiliations:** 1 Department of Neuroimmunology, Research Institute of Environmental Medicine, Nagoya University, Nagoya, Japan; 2 Futuristic Environmental Simulation Center, Research Institute of Environmental Medicine, Nagoya University, Nagoya, Japan; National Center for Geriatrics and Gerontology, Japan

## Abstract

Soluble oligomeric amyloid β (oAβ) causes synaptic dysfunction and neuronal cell death, which are involved in the pathogenesis of Alzheimer's disease (AD). The hematopoietic growth factor granulocyte-colony stimulating factor (G-CSF) is expressed in the central nervous system (CNS) and drives neurogenesis. Here we show that G-CSF attenuated oAβ neurotoxicity through the enhancement of the enzymatic activity of Aβ-degrading enzyme neprilysin (NEP) in neurons, while the NEP inhibitor thiorphan abolished the neuroprotection. Inhibition of MEK5/ERK5, a major downstream effector of G-CSF signaling, also ablated neuroprotective effect of G-CSF. Furthermore, intracerebroventricular administration of G-CSF enhanced NEP enzymatic activity and clearance of Aβ in APP/PS1 transgenic mice. Thus, we propose that G-CSF may be a possible therapeutic strategy against AD.

## Introduction

Alzheimer's disease (AD) is a neurodegenerative disorder and the most common cause of dementia in the elderly. One of the pathological hallmarks of AD is senile plaque, whose major component is fibrillar amyloid β (fAβ). While fAβ-induces neuronal dystrophy and tau hyperphosphorylation [1], [2], soluble oligomeric Aβ (oAβ) has been reported to exhibit higher neurotoxicity than fAβ. oAβ reportedly inhibits hippocampal long-term potentiation and disrupts synaptic plasticity [3], [4].

Granulocyte-colony stimulating factor (G-CSF) is a major growth factor in the differentiation and proliferation of neutrophilic-granulocytic lineage cells that modulates the immune response by inhibiting the production of inflammatory cytokines [5], [6]. Both G-CSF and its receptor G-CSFR are widely expressed in neurons in the central nervous systems (CNS), and their expression is induced by ischemia [7]. G-CSFR is also reportedly expressed in adult neural stem cells, and G-CSF can induce neuronal differentiation *in vitro* [7]. However, the exact functions of G-CSF await further elucidation.

Administration of G-CSF has been shown to improve cognitive performance in an AD model mouse carrying the Tg2576 transgene without reduction of Aβ burden [8]. The mechanism is reported to be due to local neurogenesis surrounding Aβ aggregates and the enhancement of acetylcholine levels. Another report shows that G-CSF ameliorates cognitive impairments with accompanying decreases of Aβ burden in APP/PS1 transgenic (Tg) mouse model of AD [9]. The study reported that the effects of G-CSF are due to upregulation of neurogenesis by neuronal stem cells and Aβ clearance by microglia. However, the precise functions of G-CSF on mature neurons are not fully understood. Increasing zinc-metalloprotease neprilysin (NEP) activity in AD mouse models reportedly improves cognitive impairments [10]. Indeed, NEP is one of the most prominent Aβ degrading enzymes. In this study, we show that G-CSF attenuates oAβ_1–42_ toxicity via activation of NEP.

## Materials and Methods

### Preparation of oligomeric Aβ_1–42_

Soluble oligomeric amyloid β_1–42_ (oAβ_1–42_) was prepared as described previously [11]. Briefly, synthetic human Aβ_1–42_ (Peptide Institute, Osaka, Japan) was dissolved in 100% 1,1,1,3,3,3-hexafluoro-2-propanol at a concentration of 1 mM. This solution was completely dried by the vacuum desiccator. The obtained film was resuspended in dimethyl sulfoxide to a concentration of 5 mM, and diluted with Dulbecco's Modified Eagle Medium/F12 (Invitrogen, Carlsbad, CA, USA) at a concentration of 100 µM. This solution was incubated at 4°C for 24 h to obtain oAβ_1–42_. A final concentration of 5 µM oAβ_1–42_ was used in all experiments.

### Animals

This study was carried out in strict accordance with the guideline for the care and use of laboratory animals of Nagoya University. All protocols for animal experiments were approved by the Animal Experiment Committee of Nagoya University. Transgenic mice expressing mutant variants of human amyloid precursor protein (APP) with K595N and M596L mutations and presenilin 1 (PS1) with A264E mutation were purchased from the Jackson Laboratory (B6C3-Tg(APP695)3Dbo Tg(PSEN1)5Dbo/J; #003378) and were backcrossed to C57BL/6J mice for more than 10 generations after purchase (here designated as APP/PS1 Tg mice).

G-CSF (30 ng/3 µl) or vehicle [phosphate-buffered saline (PBS)] was injected into the cerebral ventricular space of 12-month-old APP/PS1 Tg mice as previously described [12], [13]. Three days after injection, deep-anesthetized mice were transcardially perfused with ice-cold PBS, and the brains were collected. The left hemispheres were used for histological analysis, and the right hemispheres were used for assessments of neprilysin enzymatic activity and Aβ concentration.

### Neuronal culture

Primary mouse cortical neurons were prepared as previously described [11], [14]. Briefly, cerebral cortices were isolated from C57BL/6J mouse embryos on the 17^th^ embryonic day, minced and treated with dissociation solution (Sumitomo Bakelite, Akita, Japan). Neurons were resuspended in Nerve Culture Medium (Sumitomo Bakelite), plated on polyethylenimine-coated glass coverslips (Asahi Techno Glass, Chiba, Japan) at a density of 5×10^3^ cells/well in 96-well multidishes, 5×10^4^ cells/well in 24-well multidishes, or 6×10^6^ cells/well in 6-well multidishes, and incubated at 37°C in an atmosphere containing 5% CO_2_ at 100% humidity. The purity of the cultures was greater than 95% based on NeuN-specific immunostaining. Neurons were used at 14 days *in vitro* for the following assessments.

### Immunocytochemistry

Neurons were plated at a density of 5×10^4^ cells per well in 24-well multidishes, and stimulated with 1–100 ng/ml G-CSF (R&D Systems) 3 h before oAβ_1–42_ stimulation. Cells were treated with 0.3–30 µM BIX02189 as an ERK5/MEK5 inhibitor (Selleck, Houston, TX, USA) or 0.1–10 µM DL-thiorphan as a neprilysin inhibitor (Enzo Life Sciences, Farmingdale, NY, USA) 1 h before G-CSF stimulation. After 24-h stimulation of oAβ_1–42_, neurons were fixed with 4% paraformaldehyde for 10 min and permeabilized with 0.1% Triton X-100 for 5 min at room temperature. After blocking with 5% goat serum for 1 h at room temperature, cells were stained with rabbit polyclonal anti-microtubule–associated protein (MAP)-2 antibody (1∶1000, Millipore, Billerica, MA, USA), and Aβ was stained with mouse monoclonal anti-Aβ antibody (clone 4G8, 1∶1000, Millipore). Images were analyzed with a deconvolution fluorescent microscope system (BZ-8000; Keyence, Osaka, Japan).

### Assessments of neuronal survival

Neuronal survival was assessed by the number of MAP-2–positive neurons and 2-(2-methoxy-4-nitrophenyl)-3-(4-nitrophenyl)-5-(2,4-disulfophenyl)-2H-tetrazolium (WST-8) assay as previously described [15]. To count MAP-2–positive neurons and normalized based on results observed with untreated neurons. Viable neurons stained strongly with an anti–MAP-2 antibody, whereas damaged neurons showed much weaker staining. The number of MAP-2–positive neurons was counted in 10 random fields per well. More than 200 cells were examined in three independent trials. The number of untreated viable neurons was normalized to 100%.

### Immunohistochemistry

Ten-micrometer-thick frozen sections of APP/PS1 Tg mouse brains were prepared using a previously described method [11]. Sections were permeabilized with 1% Triton X-100 after blocking with 10% normal goat serum for 30 min, and then were incubated with anti-Aβ mouse monoclonal antibody (clone 4G8, 1∶500, Chemicon) overnight at 4°C. After rinsing, they were incubated with Alexa488-conjugated secondary antibody (1∶1,000, Invitrogen) and 1 µg/ml Hoechst33342 for 1 h at room temperature. After rinsing, they were mounted in Fluoromount-G (SouthernBiotech). Images were analyzed with a deconvolution fluorescence microscope system (Keyence).

### RNA extraction and reverse transcription-PCR (RT-PCR)

The mRNA expression of neprilysin was detected by RT-PCR. Neurons were plated at a density of 5×10^4^ cells per well in 24-well multidishes, and stimulated with or without 100 ng/ml G-CSF (R&D Systems, Minneapolis, MN, USA) for 6 h. Total RNA was extracted from neurons using RNeasy Mini Kit (Qiagen, Valencia, CA, USA). cDNA synthesis was performed using SuperScript II (Invitrogen). PCR was carried out using the following primers.

neprilysin sense: 5′-GACCTTACTTGGATGGATGC-3′

neprilysin antisense: 5′-ACCATACACTGGGATTGGTC-3′

GAPDH sense: 5′-ACTCACGGCAAATTCAACG-3′

GAPDH antisense: 5′-CCCTGTTGCTGTAGCCGTA-3′

### Measurement of protein level and enzymatic activity of NEP

The cell membrane fractions from the mouse primary neurons or the APP/PS1 Tg mouse brains were harvested and assessed for NEP protein levels using specific ELISA (R&D Systems). NEP enzymatic activity was also examined as described previously [16]. The fluorescence of each samples were measured by a Wallac 1420 ARVO_MX_ (PerkinElmer Japan, Yokohama, Japan).

### Human Aβ ELISA

To evaluate oAβ_1–42_ in neuronal culture, we used a human Aβ oligomer specific ELISA kit (IBL, Gunma, Japan). Neurons were pre-treated with 10 µM thiorphan for 1 h and then treated with G-CSF for 3 h prior to the addition of 5 µM oAβ_1–42_ for 24 h. The neuronal culture supernatants were assessed with an ELISA kit. To evaluate the amount of human Aβ_1–40_ and Aβ_1–42_ in mouse brains, we used a human Aβ_1–40_ and Aβ_1–42_ specific ELISA kit (Wako Pure Chemical Industries, Osaka, Japan) as previously described [17]. Brains were homogenized with TNE lysis buffer [50 mM Tris-HCl at pH 7.6, 1% Nonidet P-40, 150 mM NaCl, 2 mM EDTA, and protease inhibitor mixture (Complete Mini EDTA-free, Roche, Germany)] and centrifuged at 10,000 *g* for 15 min at 4°C. The supernatants were analyzed by each Aβ specific ELISA kit. The values obtained were corrected with the wet weight of each brain sample.

### Statistical Analysis

Statistical significance was analyzed with a Student's *t*-test or one-way analysis of variance followed by Tukey's post-hoc test using GraphPad Prism version 5.0 (GraphPad Software, La Jolla, CA, USA).

## Results

### G-CSF rescues oAβ_1–42_-induced neuronal damage

**Figure 1:**
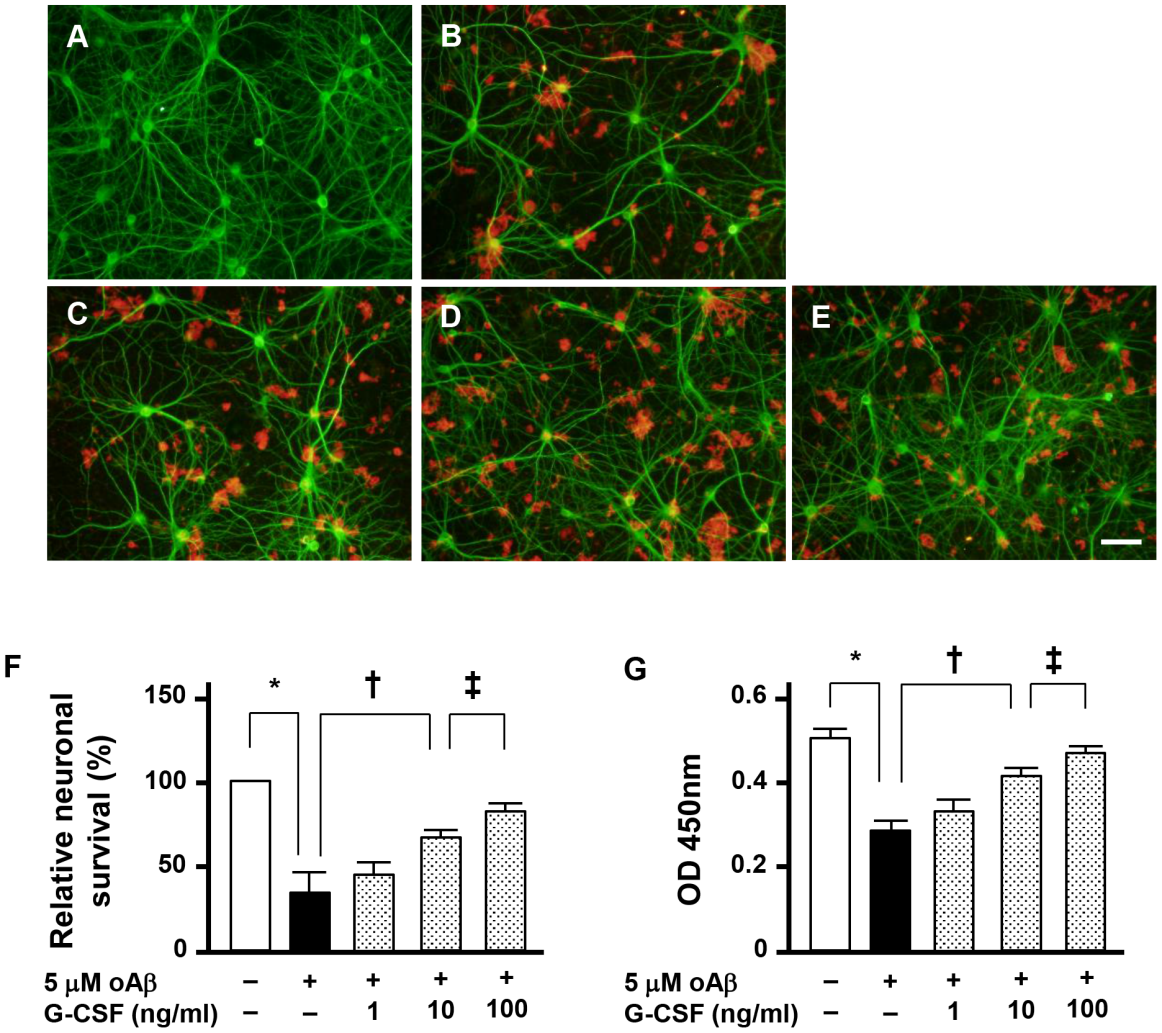
G-CSF suppresses oAβ-induced neurotoxicity. A–E, Fluorescent microscopic images of mouse primary cortical neuron cultures. A, Untreated neurons. B, Neurons treated with 5 µM oAβ_1–42_. C, Neurons treated with 5 µM oAβ_1–42_ and 1 ng/ml G-CSF. D, Neurons treated with 5 µM oAβ_1–42_ and 10 ng/ml G-CSF. E, Neurons treated with 5 µM oAβ_1–42_ and 100 ng/ml G-CSF. Treatment with G-CSF was neuroprotective against oAβ-mediated toxicity. Neurons were stained with anti–MAP-2 antibodies (green), and Aβ was stained with 4G8 antibodies (red). Scale bar: 50 µm. F, Relative neuronal survival. The number of viable neurons (MAP-2–positive neurons) was quantified relative to results observed with untreated neurons. G-CSF rescued neurons against oAβ-mediated toxicity in a dose-dependent manner. *, *p*<0.001; †, *p*<0.01; ††, *p*<0.05. Values are means ± SEM (n = 3). G, WST-8 assay. G-CSF enhanced neuronal survival against oAβ-mediated toxicity in a dose-dependent manner. *, *p*<0.001; †, *p*<0.01; ††, *p*<0.05. Values are means ± SEM (n = 6).

We first examined the effects of G-CSF on oAβ_1–42_-induced neurotoxicity using mouse primary neuronal culture (Figure 1). We found that treatment with 5 µM oAβ_1–42_ for 24 h resulted in severe neurotoxicity (Figure 1B; Figure 1F and 1G, black columns). Three hours before the addition of 5 µM oAβ_1–42_, we then applied 1–100 ng/ml G-CSF for 24 h. Treatment with G-CSF significantly suppressed oAβ_1–42_-induced neuronal damage in a dose-dependent manner (Figure 1C–E; Figure 1F and 1G, shaded columns).

### G-CSF enhances oAβ degradation through activation of NEP

**Figure 2:**
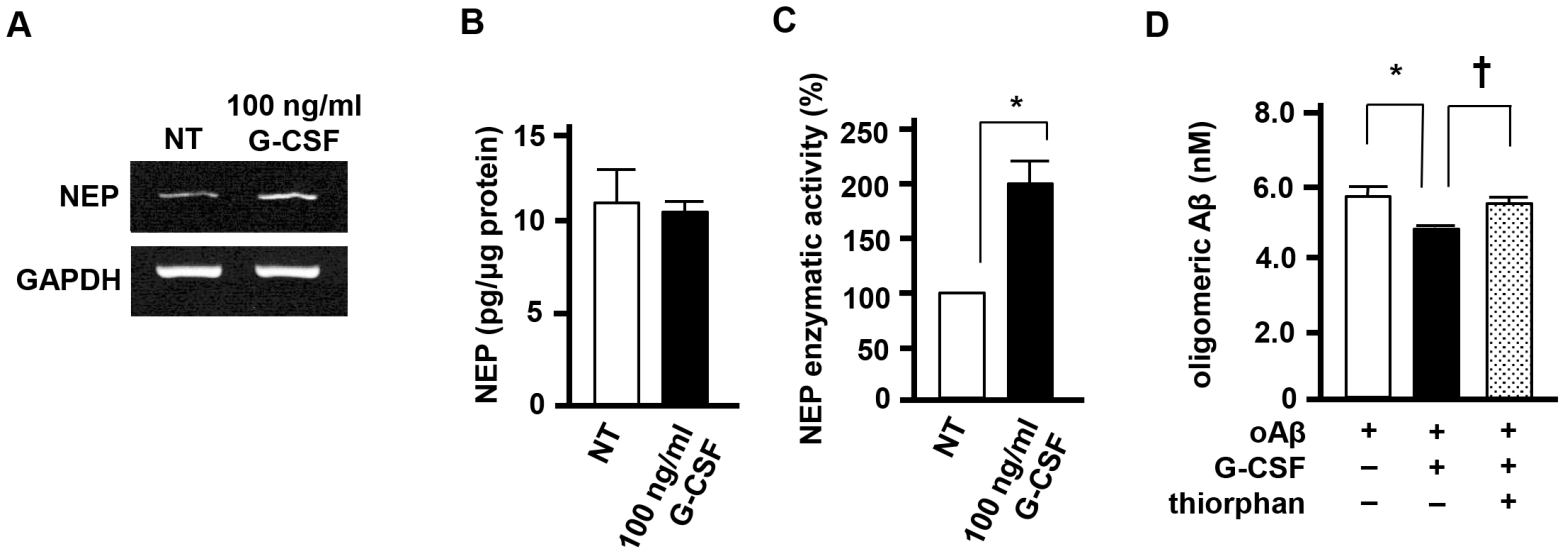
G-CSF enhances oAβ degradation by activation of NEP. A, Representative RT-PCR data for NEP in neurons. G-CSF stimulation upregulated NEP expression. NT, no treatment. B, ELISA data for NEP. NT, no treatment. Values are means ± SEM (n = 3). C, NEP enzymatic activity assay. G-CSF stimulation enhanced NEP enzymatic activity whereas NEP protein level was not affected. NT, no treatment. *, *p*<0.05. Values are means ± SEM (n = 3). D, ELISA data for oAβ. G-CSF treatment reduced the amount of oAβ_1–42_ in the supernatants of neuronal cultures, whereas the NEP inhibitor thiorphan ablated this effect. *, *p*<0.01; †, *p*<0.05. Values are means ± SEM (n = 3).

Next, we assessed whether G-CSF treatment alters the amount of Aβ applied in neuronal culture. We found that G-CSF significantly decreased Aβ concentration in neuronal culture (Figure 2D, black column). We then assessed the expression levels of Aβ-degrading enzymes [NEP and insulin-degrading enzyme (IDE)] in G-CSF–treated neurons. RT-PCR data indicated that the addition of G-CSF upregulated the expression level of NEP in neurons, whereas IDE was not affected (Figure 2A and data not shown). Next, we assessed the protein levels and enzymatic activity of NEP. G-CSF treatment significantly enhanced NEP enzymatic activity, but not NEP protein level (Figure 2B and 2C). Inhibition of NEP by thiorphan completely reversed the amount of Aβ (Figure 2D, dotted column), suggesting that Aβ degradation by G-CSF stems from the activation of neuronal NEP. Treatment with thiorphan alone did not affect the amount of Aβ.

### NEP is critical for the neuroprotective effect of G-CSF

**Figure 3:**
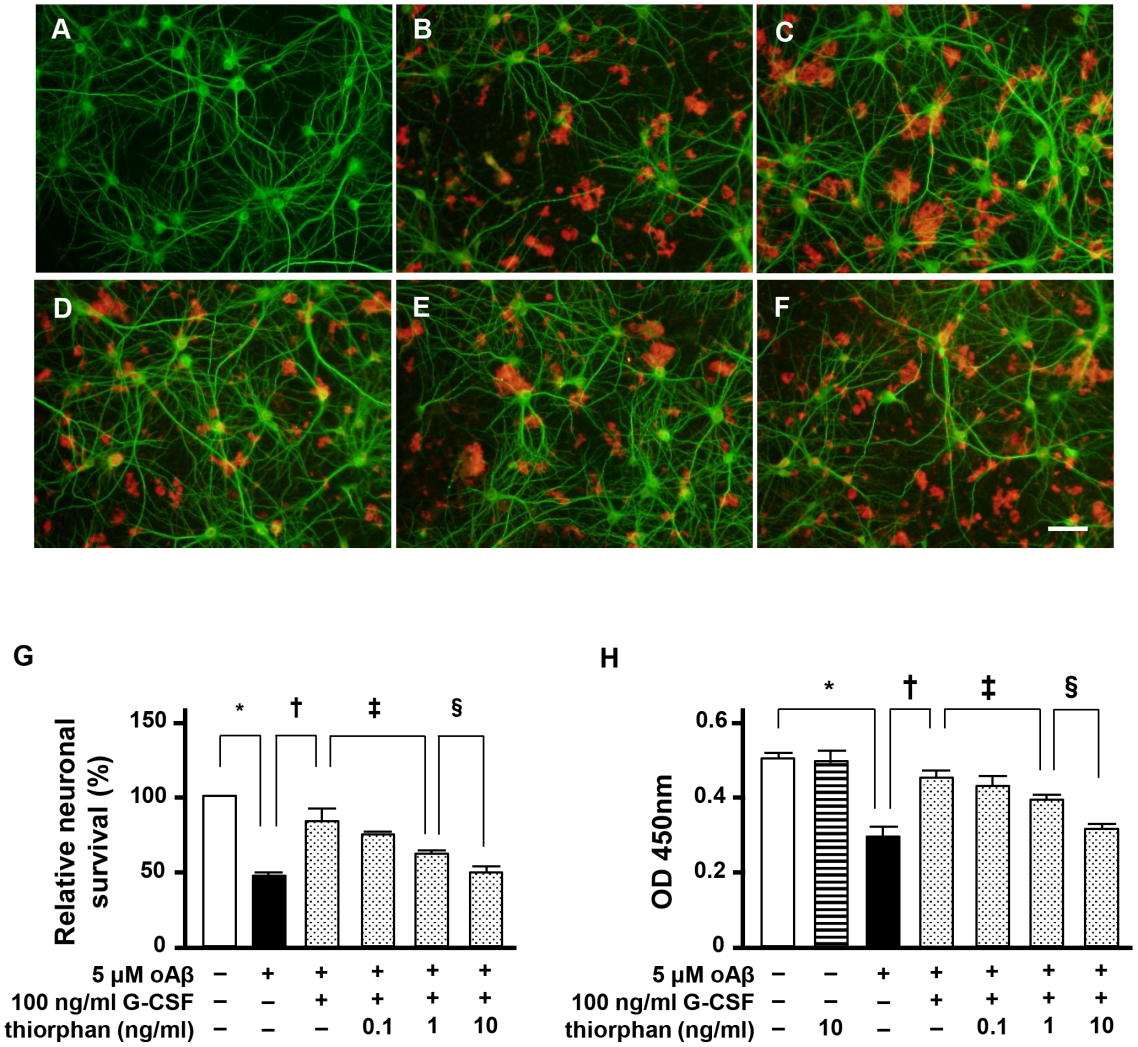
Neuroprotective effect of G-CSF depends on NEP. A–F, Fluorescent microscopic images of mouse primary cortical neuron cultures. A, Untreated neurons. B, Neurons treated with 5 µM oAβ_1–42_. C, Neurons treated with 5 µM oAβ_1–42_ and 100 ng/ml G-CSF. D, Neurons treated with 5 µM oAβ_1–42_, 100 ng/ml G-CSF and 0.1 ng/ml thiorphan. E, Neurons treated with 5 µM oAβ_1–42_, 100 ng/ml G-CSF and 1 ng/ml thiorphan. F, Neurons treated with 5 µM oAβ_1–42_, 100 ng/ml G-CSF and 10 ng/ml thiorphan. The NEP inhibitor canceled the neuroprotective effects of G-CSF. Neurons were stained with anti–MAP-2 antibodies (green) and Aβ was stained with 4G8 antibodies (red). Scale bar: 50 µm. G, Relative neuronal survival. The number of viable neurons (MAP-2–positive neurons) was quantified relative to results observed with untreated neurons. The NEP inhibitor dose-dependently suppressed the neuroprotective effects of G-CSF. *, *p*<0.001; †, *p*<0.001; ‡, *p*<0.05; §, *p*<0.01. Values are means ± SEM (n = 3). H, WST-8 assay. The NEP inhibitor reversed the neuroprotective effects of G-CSF in a dose-dependent manner. *, *p*<0.001; †, *p*<0.001; ‡, *p*<0.05; §, *p*<0.01. Values are means ± SEM (n = 6).

We assessed whether the neuroprotective effect of G-CSF results from NEP (Figure 3). We found that treatment with the NEP inhibitor thiorphan almost completely ablated the neuroprotective effects of G-CSF (Figure 3D–F; Figure 3G and 3H, shaded columns). These findings imply that treatment with G-CSF enhanced neuronal NEP activity and protected against oAβ_1–42_-induced neurotoxicity through Aβ degradation.

### The MEK5/ERK5 pathway contributes to G-CSF–mediated neuroprotection

**Figure 4:**
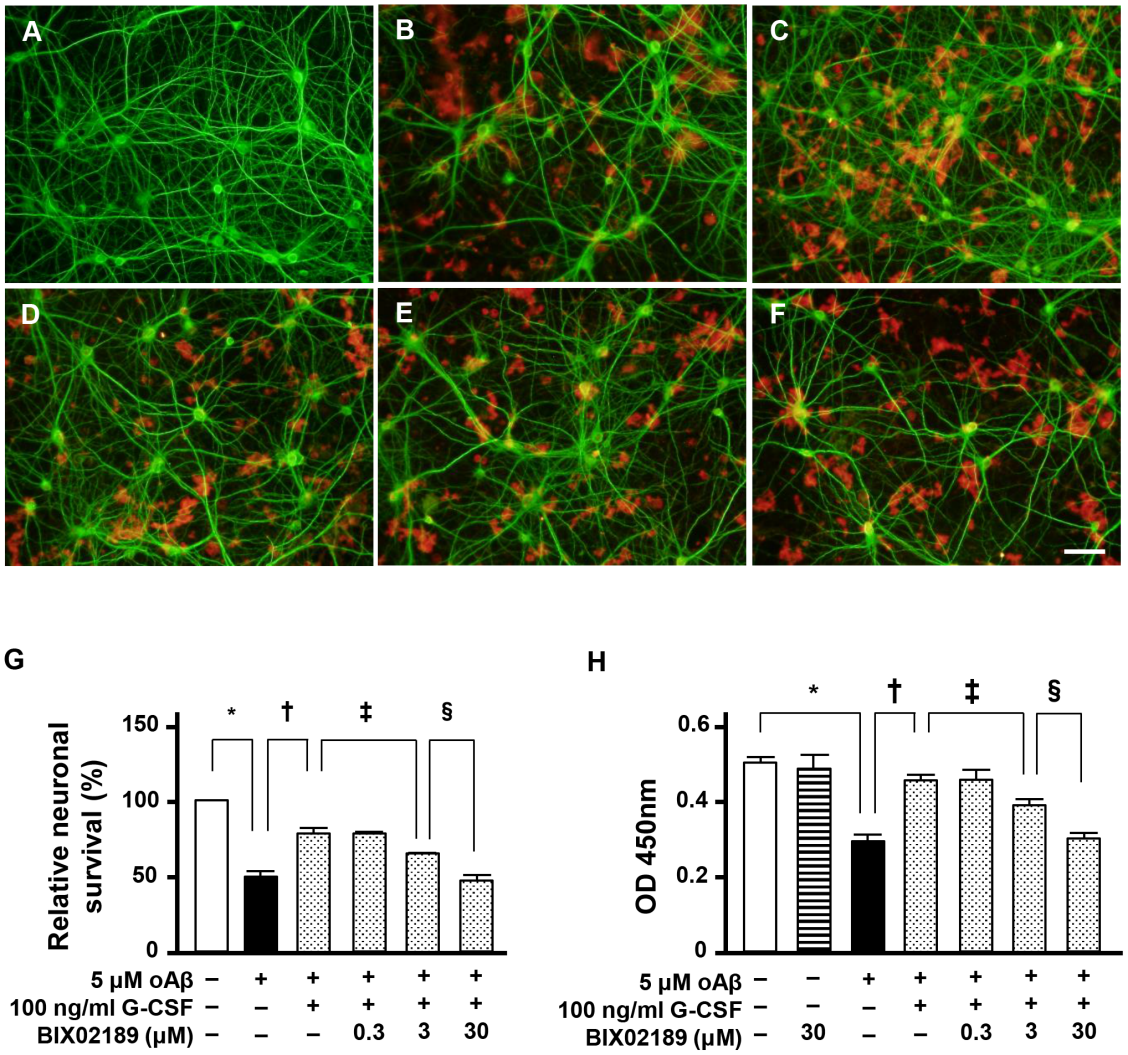
Neuroprotective effect of G-CSF requires MEK5/ERK5 signaling. A–F, Fluorescent microscopic images of mouse primary cortical neuron cultures. A, Untreated neurons. B, Neurons treated with 5 µM oAβ_1–42_. C, Neurons treated with 5 µM oAβ_1–42_ and 100 ng/ml G-CSF. D, Neurons treated with 5 µM oAβ_1–42_, 100 ng/ml G-CSF and 0.3 µM BIX02189. E, Neurons treated with 5 µM oAβ_1–42_, 100 ng/ml G-CSF and 3 µM BIX02189. F, Neurons treated with 5 µM oAβ_1–42_, 100 ng/ml G-CSF and 30 µM BIX02189. The MEK5/ERK5 inhibitor BIX02189 almost completely suppressed G-CSF–mediated protection against oAβ-induced neurotoxicity. Neurons were stained with anti–MAP-2 antibodies (green), and Aβ was stained with 4G8 antibodies (red). Scale bar: 50 µm. G, Relative neuronal survival. The number of viable neurons (MAP-2–positive neurons) was quantified relative to results observed with untreated neurons. Inhibition of MEK5/ERK5 almost completely ablated the neuroprotective effects of G-CSF. *, *p*<0.001; †, *p*<0.001; ‡, *p*<0.001. Values are means ± SEM (n = 3). H, WST-8 assay. Blocking MEK5/ERK5 almost completely canceled the neuroprotective effects of G-CSF. *, *p*<0.001; †, *p*<0.001; ‡, *p*<0.001. Values are means ± SEM (n = 6).

The MEK5/ERK5 pathway is a major downstream effector of G-CSF signaling. We examined the role of the MEK5/ERK5 pathway in G-CSF–mediated neuroprotection. We found that inhibition of MEK5/ERK5 by BIX02189 almost completely suppressed the neuroprotective effects of G-CSF against oAβ-induced neurotoxicity (Figure 4D–F; Figure 4G and 4H, shaded columns). We confirmed BIX02189 decreased NEP activity in G-CSF-treated neurons. These results suggest that G-CSF–mediated neuroprotection depended on MEK5/ERK5 signaling.

### *In vivo* G-CSF treatment enhances Aβ_1–42_ degradation by activation of NEP

**Figure 5:**
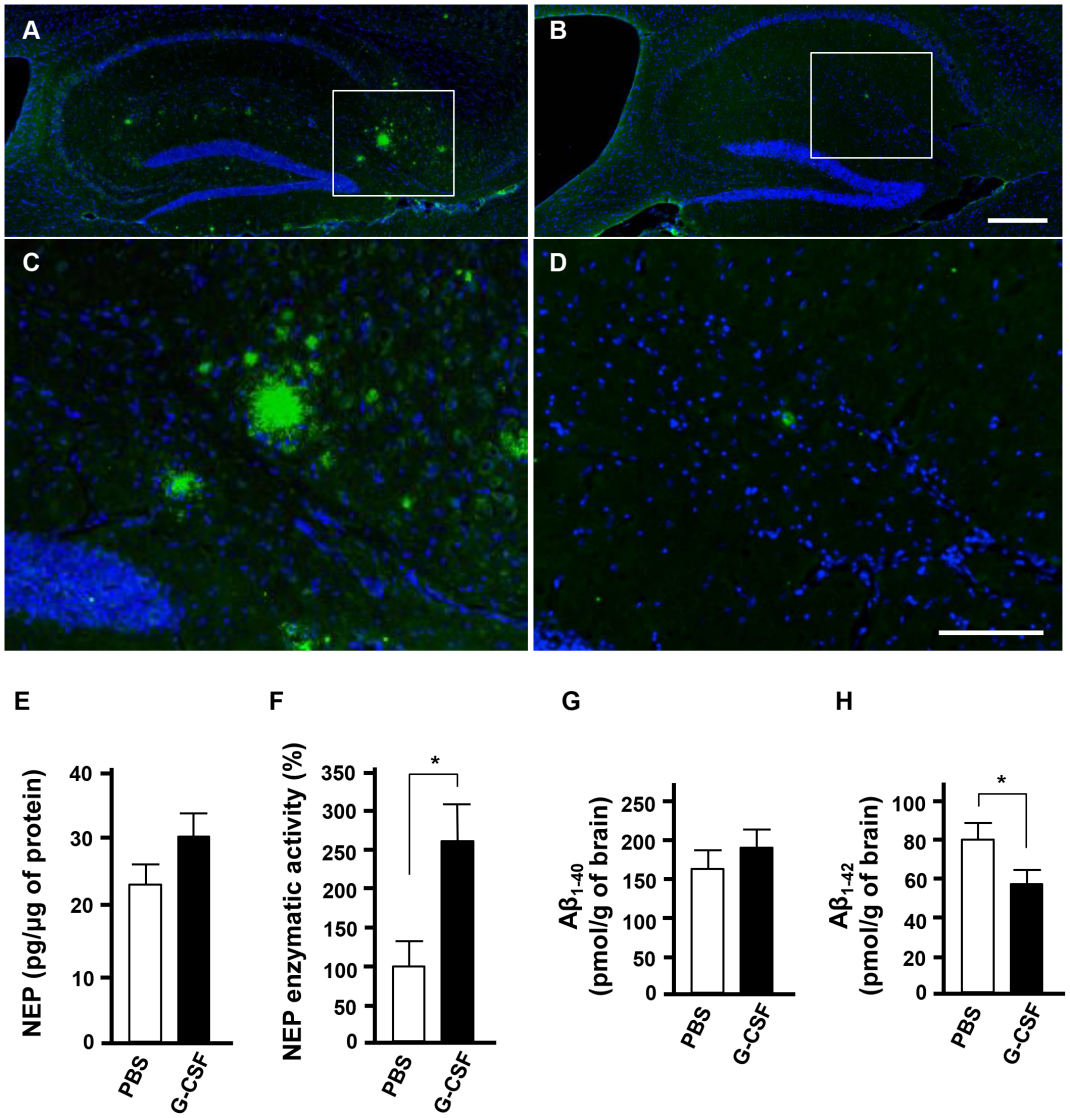
*In vivo* G-CSF treatment enhances Aβ_1–42_ degradation by activation of NEP. A–D, Fluorescent microscopic images of hippocampi from 12-month-old APP/PS1 Tg mice. A, PBS-injected APP/PS1 Tg mice. B, G-CSF–injected APP/PS1 Tg mice. C, Higher magnification image of the white-framed area in A. D, Higher magnification image of the white-framed area in B. Green, Aβ (4G8); blue, nucleus (Hoechst). Scale bar; 200 µm in A and B, 100 µm in C and D. E, ELISA data for NEP. Values are means ± SEM (n = 3). F, NEP enzymatic activity assay. G-CSF treatment enhanced NEP enzymatic activity in APP/PS1 Tg mouse brains, whereas NEP protein levels were not affected. *, *p*<0.05. Values are means ± SEM (n = 3). G, ELISA data for human Aβ_1–40_ in APP/PS1 Tg mouse brains. H, ELISA data for human Aβ_1–42_ in APP/PS1 Tg mouse brains. G-CSF treatment significantly reduced Aβ_1–42_ load, but not Aβ_1–40_, in APP/PS1 Tg mice. *, *p*<0.05. Values are means ± SEM (n = 3).

Finally, we examined whether G-CSF treatment enhances NEP activity and Aβ degradation using APP/PS1 Tg mice, a mouse model of Alzheimer's disease. G-CSF was injected into the cerebral ventricular space of APP/PS1 mice. Three days after injection, mouse brains were assessed by histological and biochemical analysis. Histological analysis revealed that G-CSF treatment reduced the Aβ burden in the hippocampus, whereas PBS-treated mice showed substantial amounts of Aβ deposits (Figure 5A–D). As expected, G-CSF treatment significantly enhanced NEP activity in the brains of APP/PS1 Tg mice, whereas NEP protein levels were not affected (Figure 5E and 5F). Human Aβ-specific ELISAs also revealed that G-CSF injection significantly reduced the amount of Aβ_1–42_ in APP/PS1 transgenic mice, whereas Aβ_1–40_ load was not affected (Figure 5G and 5H).

## Discussion

The present study revealed a novel neuroprotective function of G-CSF against oAβ toxicity. We found that G-CSF significantly enhanced neuronal NEP activity and led to increased degradation of oAβ. Furthermore, injection of G-CSF into the cerebral ventricular space of APP/PS1 mice also enhanced oAβ degradation by activation of NEP.

NEP is the major Aβ degrading peptidase. NEP deficiency results in elevated levels of endogenous Aβ_1–40_ and Aβ_1–42_ in the hippocampus, cortex, thalamus/striatum, and cerebellum [18]. NEP is also reported to degrade Aβ_1–40_ more rapidly than Aβ_1–42_ *in vitro* [19]. However, our in *vivo* data show that G-CSF reduced the amount of Aβ_1–42_ in APP/PS1 Tg mice, though Aβ_1–40_ was not affected. Clearance of Aβ_1–40_ may not depend on NEP-catalyzed proteolysis as that of Aβ_1–42_. These results suggest that G-CSF has an effect on Aβ_1–42_ degradation via NEP activation *in vivo*. While NEP is capable of cleaving Aβ monomers, its ability to degrade oAβ is controversial [10]. However, a recent report shows that NEP gene transfer into an AD mouse model significantly reduces oAβ [20]. In the present study, we have shown that G-CSF treatment reduced the amount of oAβ in the supernatants of neuronal cultures via activation of NEP. Therefore, NEP is clearly able to degrade oAβ.

Another Aβ degrading enzyme, IDE, is reported to be reduced in the hippocampus of AD [21]. The enhanced IDE activity in IDE and APP double-transgenic mice decreases Aβ levels and prevents the formation of AD pathology. However, G-CSF did not induce activation of IDE in neurons in that study. The reduced level of oAβ was small. Other mechanism such as neurogenesis may be involved in neuroprotection.

G-CSF activates the Jak/Stat, MAPK (Erk1/2, JNK and p38), PI3-K, and Src family kinase cascades [22]. A recent study shows that the MEK5/ERK5 pathway is a major downstream effector of G-CSF signaling in the regulation of cell proliferation and survival. [23], [24]. In the present study, inhibition of MEK5/ERK5 by BIX02189 almost completely suppressed the neuroprotective effects of G-CSF against oAβ-induced neurotoxicity. The results suggest that G-CSF–induced NEP is activated by the MEK5/ERK5 pathway. MEK5/ERK5 pathway is involved in cell proliferation, cell survival, and angiogenesis. However, the precise mechanism of NEP expression by MEK5/ERK5 remains unknown.

The G-CSF receptor is also expressed in microglia, and expression is increased after spinal cord injury [25]. G-CSF has been shown to promote the recruitment of microglia to the injury site, which regulates microglial activation by inhibiting the activity of NF-κB. [26]. In the previous study, G-CSF increased microglial burden, reduced Aβ deposition, and ameliorated the cognitive impairments in APP/PS1 mice. This mechanism is considered to be microglial Aβ clearance and neurogenesis in neural stem cells [9]. Therefore, microglial Aβ clearance may also contribute to decreasing the amount of Aβ_1–42_ by G-CSF injection in APP/PS1 transgenic mice in the present study. Taken together, the present study shows that G-CSF significantly enhances the expression level and enzymatic activity of NEP in neurons, indicating that G-CSF could be a useful therapeutic strategy against oAβ_1–42_ neurotoxicity in AD.
